# Preclinical and clinical pharmacology of brexanolone (allopregnanolone) for postpartum depression: a landmark journey from concept to clinic in neurosteroid replacement therapy

**DOI:** 10.1007/s00213-023-06427-2

**Published:** 2023-08-11

**Authors:** Doodipala Samba Reddy, Robert H. Mbilinyi, Emily Estes

**Affiliations:** 1 Department of Neuroscience and Experimental Therapeutics, Texas A&M University School of Medicine, Bryan, TX 77807 USA; 2 Institute of Pharmacology and Neurotherapeutics, Texas A&M University Health Science Center, 8447 Riverside Pkwy, Bryan, TX 77807 USA

**Keywords:** Allopregnanolone, Antidepressant, Brexanolone, GABA-A receptor, Postpartum depression

## Abstract

This article describes the critical role of neurosteroids in postpartum depression (PPD) and outlines the landmark pharmacological journey of brexanolone as a first-in-class neurosteroid antidepressant with significant advantages over traditional antidepressants. PPD is a neuroendocrine disorder that affects about 20% of mothers after childbirth and is characterized by symptoms including persistent sadness, fatigue, dysphoria, as well as disturbances in cognition, emotion, appetite, and sleep. The main pathology behind PPD is the postpartum reduction of neurosteroids, referred to as neurosteroid withdrawal, a concept pioneered by our preclinical studies. We developed neurosteroid replacement therapy (NRT) as a rational approach for treating PPD and other conditions related to neurosteroid deficiency, unveiling the power of neurosteroids as novel anxiolytic-antidepressants. The neurosteroid, brexanolone (BX), is a progesterone-derived allopregnanolone that rapidly relieves anxiety and mood deficits by activating GABA-A receptors, making it a transformational treatment for PPD. In 2019, the FDA approved BX, an intravenous formulation of allopregnanolone, as an NRT to treat PPD. In clinical studies, BX significantly improved PPD symptoms within hours of administration, with tolerable side effects including headache, dizziness, and somnolence. We identified the molecular mechanism of BX in a neuronal PPD-like milieu. The mechanism of BX involves activation of both synaptic and extrasynaptic GABA-A receptors, which promote tonic inhibition and serve as a key target for PPD and related conditions. Neurosteroids offer several advantages over traditional antidepressants, including rapid onset, unique mechanism, and lack of tolerance upon repeated use. Some limitations of BX therapy include lack of aqueous solubility, limited accessibility, hospitalization for treatment, lack of oral product, and serious adverse events at high doses. However, the unmet need for synthetic neurosteroids to address this critical condition supersedes these limitations. Recently, we developed novel hydrophilic neurosteroids with a superior profile and improved drug delivery. Overall, approval of BX is a major milestone in the field of neurotherapeutics, paving the way for the development of novel synthetic neurosteroids to treat depression, epilepsy, and status epilepticus.

**Graphical abstract:**

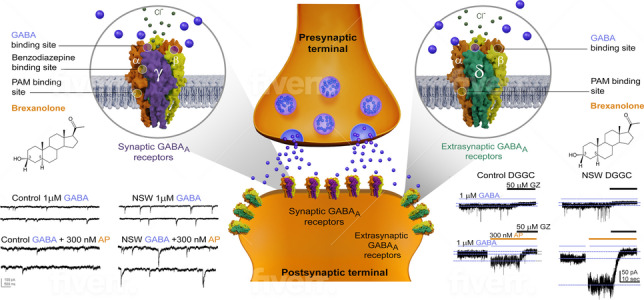

## Introduction

Postpartum depression (PPD) is a major depressive episode with onset after childbirth or within 4 weeks of delivery. This neuroendocrine condition affects ~20% of mothers in the USA (Meltzer-Brody et al. 2018a; Bauman et al. 2020). Clinical diagnosis of PPD is based on the Diagnostic and Statistical Manual of Mental Disorders (DSM-V) criteria for a major depressive episode with onset of symptoms in the third trimester or within 4 weeks of delivery. Like major depression, PPD is characterized by sadness, anhedonia, and mood symptoms including cognitive impairment, feelings of worthlessness or guilt, and suicidal ideation. Many PPD cases arise directly after childbirth, although around 50% of patients experience symptoms before delivery (Yonkers et al. 2001; Leader et al. 2019). Due to a lack of provider awareness and negative social stigma attached to PPD, patients often go underdiagnosed and untreated. Consequently, the available data on PPD prevalence likely reflects a small subset of the patients suffering from this condition (Pinto-Foltz and Logsdon 2008; Sudhanthar et al. 2019).

PPD has a devastating impact on the well-being of both mother and infant, with lasting consequences, including significant repercussions on mother-infant attachment, infant nutrition, and well-being, and complete family functioning. Because PPD can hinder breastfeeding, it disrupts the mother-infant bond, affecting their emotional connection and responsiveness. Infants may also experience disrupted nutrition, emotional development, and overall caregiving environment, resulting in inconsistent routines, reduced stimulation, and impaired interactions with their mothers. In severe cases, PPD can lead to neglect of the infant or thoughts of harming the infant. Additionally, due to risk of suicide, PPD is considered a life-threatening condition. Addressing PPD promptly is essential to foster healthy development and well-being of the mother and her infant, as well as the whole family.

The exact pathophysiology of PPD is unclear but is associated with a rapid decline in reproductive hormone levels and complex changes in neurotransmitters, steroid hormones, receptor signaling, and genetic predisposition (Magiakou et al. 1996; Maurer-Spurej et al. 2007; Bunevicius et al. 2009; Doornbos et al. 2009). Few effective therapies exist specifically for PPD (Brown et al. 2021) and risk factors include a personal or family history of depression during pregnancy or after giving birth. Although traditional antidepressants are non-specific therapeutic options for PPD, antidepressants take 4–8 weeks to produce a therapeutic effect, often requiring chronic dosing to elicit a treatment response (Molyneaux et al. 2014; Cox et al. 2016; Bhattacharjee et al., 2020). In PPD, this delayed therapeutic benefit puts the mother and infant at high risk. Thus, there is a compelling medical need for targeted PPD drugs that can provide rapid, efficacious relief of depressive symptoms.

**Fig. 1:**
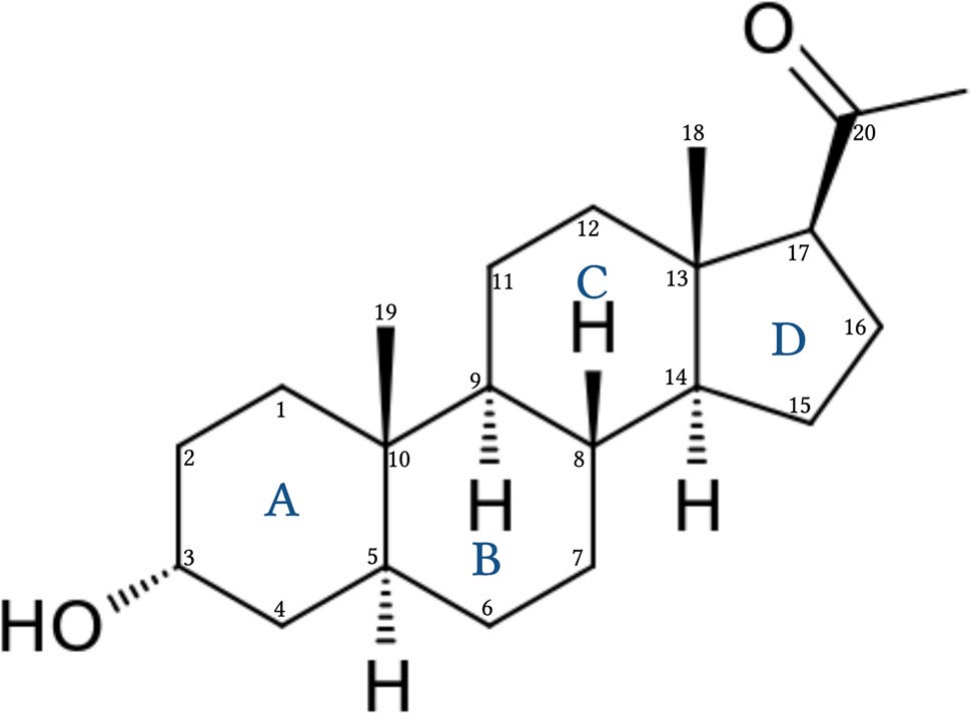
Chemical structure of brexanolone (allopregnanolone)

Neurosteroids play a key role in women’s health (Reddy 2009, 2022), with the neurosteroid, allopregnanolone (AP) being a leading compound in neurological and psychiatric studies linked to hormone alterations (Reddy and Kulkarni 1997a; Reddy et al. 2012; Reddy and Rogawski 2000a; Reddy 2022). The postpartum decline of progesterone and neurosteroids after delivery, referred to as neurosteroid withdrawal, is the currently accepted etiology for PPD (Reddy 2022). Progesterone levels peak during the third trimester, leading to elevated AP in the brain. This elevation is followed by reduced tonic inhibition currents from extracellular GABA-A receptors (Reddy 2013b; Jarman et al. 2020). After parturition, neurosteroid levels rapidly return to pre-pregnancy levels, but GABA-A receptors take longer to recover. The resulting imbalance between neurosteroid levels and GABA-A receptors may play a crucial role in PPD development (Maguire and Mody 2008; Meltzer-Brody 2011; Meltzer-Brody and Kanes 2020). The FDA approved brexanolone (BX) use in 2019, as an aqueous formulation of AP (Fig. 1) to treat PPD. Because BX therapy rapidly restores AP levels in the brain to levels similar to the third trimester of pregnancy, it has become a valuable drug to mitigate the debilitating symptoms of PPD (Reddy 2023).

This article describes neuroendocrine insights into the therapeutic use of neurosteroids for PPD, leveraging the primary concept of neurosteroid replacement therapy for PPD and other neuroendocrine conditions. We present a chronological and comprehensive synopsis of preclinical and clinical research leading to the development and approval of the neurosteroid BX (AP) for PPD. We also explain the pharmacological basis of BX as a rapid-acting antidepressant for PPD and emphasize its distinct profile over traditional antidepressants as well as a few constraints for its widespread clinical use. We outline future developments including the discovery and launch of hydrophilic neurosteroids and oral products.

## Therapeutic landscape of postpartum depression

### Postpartum depressive disorders

PPD typically manifests as depressed mood, diminished interest or pleasure in activities, change in body weight, insomnia, fatigue, psychomotor agitation or retardation, feelings of guilt, inability to concentrate, and in severe cases, suicidal ideation (Payne and Maguire 2019). Globally, PPD affects approximately 10–20% of women who have given birth. Similarly, in the USA, the Centers for Disease Control and Prevention estimates that the prevalence of PPD among new mothers varies from ~10 to 20% by state, with an average of 11.5%. Postpartum depressive disorders typically manifest in one of three clinical syndromes (postpartum blues, PPD, and postpartum psychosis) with varying severity. *Postpartum blues* (PB), the most benign of these three, refers to the development of low mood and mild depressive symptoms between 2 and 5 days postpartum, which are self-limiting, lasting less than 2 weeks (Stewart and Vigod 2016). The prevalence of PB is 70–80%, but due to its mild symptoms, treatment is not required. In contrast, PPD is persistent and causes moderate to severe dysfunction. PPD is a subtype of major depressive disorder occurring in 15–20% of mothers, with onset after delivery or within 4 weeks or months (Stewart and Vigod 2019). To meet the criteria for PPD, patients must report depressed mood or anhedonia for at least 2 weeks. Patients may also report sleep disturbance, feelings of guilt or worthlessness, changes in appetite, loss of energy, decreased concentration, and suicidal ideation (Pearlstein et al. 2009). Women who have experienced a previous PPD episode are at higher risk, with approximately 25% of them experiencing a subsequent episode. Women with PPD have an increased risk of suicide and impairments in daily function, including maternal-infant bonding. *Postpartum psychosis* (PP) is the most severe disorder on the spectrum of postpartum disorders. The onset of PP is typically sudden, often occurring within the first 2 weeks after delivery (Osborne 2018; Spinelli 2021). Due to the elevated risk of suicide and infanticide, PP is a psychiatric emergency that requires inpatient hospitalization until physicians determine that the patient is no longer a risk to themselves or the child (Wilkinson et al. 2017).

PPD is often underdiagnosed and untreated in women across many countries, in part likely due to limited screening guidelines. Recent systematic review and meta-analyses identified many PPD risk factors, including younger age, family history of affective disorders, emotional trauma, specific ethnic backgrounds, lower socioeconomic status, and presence of comorbid conditions (Zhao and Zhang 2020). PPD affect an estimated 500,000 women in the USA annually. The prevalence of PPD is projected to be high, especially in developing countries (Liu et al. 2022). The COVID-19 pandemic is known to have exacerbated psychological problems including depression and anxiety in high-risk populations, and meta-analyses specifically showed an increased PPD prevalence in pregnant women during the postpartum period (Usmani et al. 2021; Safi-Keykaleh et al. 2022). Thus, it is likely that the COVID-19 pandemic had a devastating global impact on women with postpartum conditions.

### Diagnosis of PPD

Diagnosis of PPD is based on DSM-5 diagnostic criteria, defined by the onset of a major depressive episode, depressive symptoms, and/or mood disorder during the postpartum period with onset after childbirth and within 4 weeks of delivery, although some experts extend this to 1 year postpartum. There is no separate diagnosis for PPD. According to the DSM-5, PPD patients are diagnosed using the criteria for a major depressive episode and the criteria for peripartum onset. Screening of patients and medication outcomes are evaluated using the Hamilton Rating Scale for Depression (HAM-D), a validated scale and accepted regulatory endpoint used to assess PPD, and its recovery. After evaluating mood, feelings of guilt, suicide ideation, insomnia, agitation, anxiety, weight loss, and somatic symptoms, women within the third trimester or within the first 4 weeks postpartum may be diagnosed with moderate PPD, HAM-D total score of 20–25) or severe PPD (HAM-D total score of > 26). The Edinburgh Postnatal Depression Scale (EPDS) is a self-report screening tool consisting of ten items (Cox et al. 1987). It is commonly used by healthcare professionals to identify and assess postpartum depression in mothers. It has been used during both pregnancy and the postpartum period. Higher scores indicate a greater likelihood of depression, prompting further evaluation and potential treatment.

### Therapeutic options of PPD

Prior to BX approval in 2019, no specific drugs were available to treat PPD. According to the National Institute of Mental Health, current standard PPD treatments typically involve (a) psychotherapy, and/or (b) pharmacology therapy. Psychotherapies for PPD include cognitive therapy, behavioral therapy, and supportive counseling (O’Hara and Engeldinger 2018; Šebela et al. 2019). Treatment choice depends on symptom severity and level of functional impairment. For mild to moderate PPD, psychotherapy alone is often recommended as the initial treatment. In cases of more severe symptoms, a combination of psychotherapy and medication may be used. Antidepressants are the common pharmacological therapy for PPD (Nemeroff 2008; Kroska and Stowe 2020). Most antidepressants target serotonergic and noradrenergic pathways using selective serotonin reuptake inhibitors (SSRIs) and serotonin-norepinephrine reuptake inhibitors (SNRIs). SSRIs have been a first-line therapy when psychotherapy alone is not effective (Tolliday 2021). Patients typically require treatment for 6 months to 1 year and must undergo a taper period to avoid withdrawals. However, there is little evidence that antidepressants are effective for PPD (Betcher and Wisner 2020) and antidepressants are not specifically approved by the FDA to treat PPD. The onset of antidepressant effectiveness in alleviating depressive symptoms can take weeks to months, and no substantial evidence supports the superiority of one SSRI over another in treating PPD. In addition, the risks of using such antidepressants during breastfeeding need to be carefully evaluated for both the mother and the baby.

Other treatments for PPD, including electroconvulsive therapy (ECT), repetitive transcranial magnetic stimulation (rTMS), and psychotherapy, are also used. Of note, all available treatments for depression require a significant amount of time to take effect. Antidepressant drugs, for instance, typically take around 4 weeks to show efficacy. ECT requires two sessions per week for 4–5 weeks, while rTMS is administered daily for 4 to 6 weeks. Psychotherapy typically involves attending 8–20 weekly sessions. In 2019, brexanolone was the first rapid-acting neurosteroid to receive FDA approval specifically to treat PPD. BX offers an alternative to SSRIs as a first-line PPD treatment in certified clinics. This neurosteroid stands out due to its rapid onset of action, high response rate, and minimal side effects.

## Role of neurosteroids in postpartum depression

**Fig. 2:**
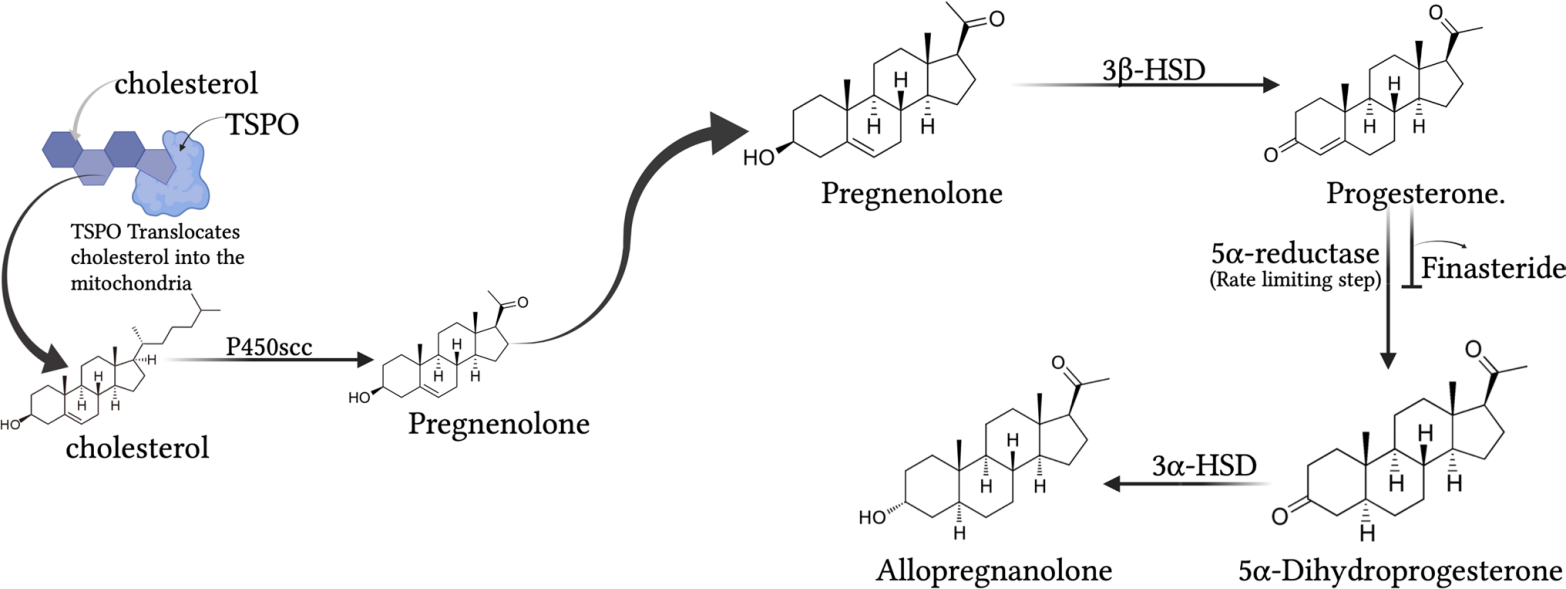
Biosynthesis of the neurosteroid allopregnanolone (brexanolone) in the brain. In neurons and glial cells, P450scc enzyme in the mitochondria converts cholesterol to pregnenolone. Pregnenolone is then transported outside of the mitochondria, where it is converted into progesterone by 3-β-hydroxysteroid dehydrogenase (3β-HSD). 5α-Reductase converts progesterone to 5α-dihydroprogesterone in the reaction’s rate-limiting step, which can be inhibited by finasteride (a 5α-reductase inhibitor). Synthesis is completed by converting dihydroprogesterone to allopregnanolone via 3α-hydroxysteroid oxidoreductase (3α-HSOR). Allopregnanolone is also synthesized from circulating progesterone from ovarian sources, such as during menstrual cycle and pregnancy

Neurosteroids affect brain function by rapidly altering neuronal excitability and affecting neuronal networks (Reddy 2009, 2022; Rupprecht and Holsboer 1999; Reddy 2003a; Do Rego et al. 2009; Gunduz-Bruce et al. 2022). This is accomplished by allosteric modulation of GABA-A receptors, the primary receptors that regulate rapidly inhibit neural networks in the brain. Although the precise mechanism is not well understood, the ability of neurosteroids to modulate GABA-A receptors is thought to occur via direct binding and increased channel conductance (Akk et al. 2001; Reddy and Rogawski 2010). Neurosteroids can be classified into three structural groups: pregnanes (e.g., allopregnanolone), androstanes (e.g., androstanediol), and sulfated neurosteroids (Reddy 2010). Pregnanes and androstanes positively modulate GABA-A receptors, whereas sulfated neurosteroids can act as negative modulators (Fig. 2). Neuroactive steroids include natural and synthetic agents that rapidly alter neuronal excitability and can have inhibitory or excitatory properties (Table 1). We are among the first to design and implement neurosteroid-based therapies to treat neuronal excitability disorders (Reddy 2003b, 2010, 2016, 2019, 2022, 2023).

**Table 1:** Pharmacological summary outline of endogenous neurosteroids

*Anxiolytic (inhibitory) neurosteroids*	*Anxiogenic (excitatory) steroids*
Progesterone	Estradiol
Allopregnanolone (brexanolone)	Pregnenolone sulfate
Pregnanolone	Dehydroepiandrosterone sulfate
Dihydroprogesterone	Cortisol
Androstanediol	11-Deoxycortisol
Etiocholanone	
Dihydrotestosterone	
Deoxycorticosterone	
Dihydrodeoxycorticosterone	
Allotetrahydrodeoxycorticosterone	

**Fig. 3:**
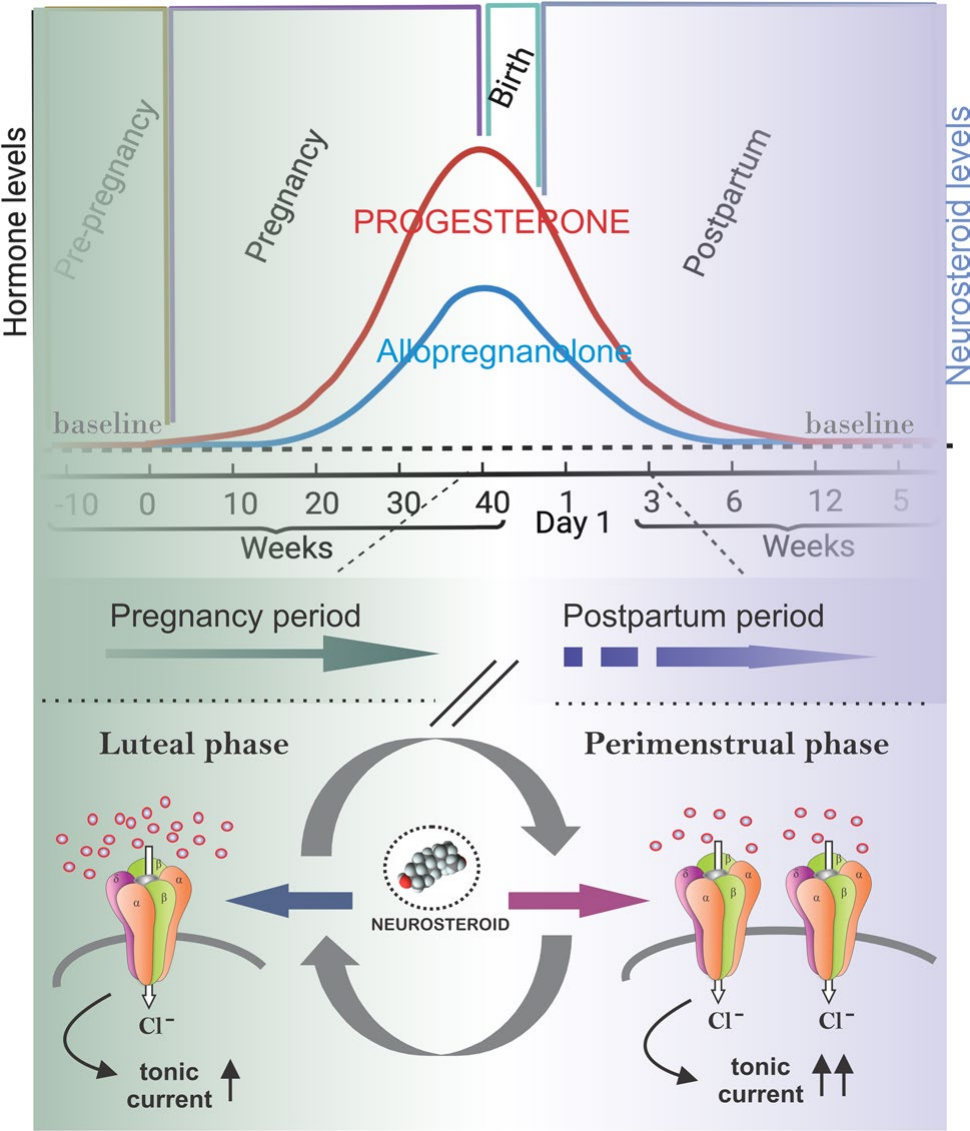
Fluctuations in progesterone and neurosteroid levels during pregnancy and postpartum period and associated changes in GABA-A receptor function. (Top panel) Temporal changes in steroid hormones and neurosteroids during pregnancy and postpartum period. Progesterone levels increase during pregnancy, peaking at the third trimester and falling to pre-pregnancy levels after parturition. When allopregnanolone (AP) levels rise, the brain responds by reducing the number of extrasynaptic GABA-A receptors, causing reduced tonic inhibition. After pregnancy, AP levels fall, resulting in addition of extrasynaptic GABA-A receptors lost during pregnancy. This process, which is thought to occur more slowly in postpartum women, could trigger PPD. (Bottom panel) GABA-A receptor plasticity and neurosteroid withdrawal model of postpartum changes. The GABA-A receptor plasticity model suggests that cyclical changes in neurosteroid levels, such as AP, during ovarian cycle phases result in altered extrasynaptic GABA-A receptor composition and abundance. Upregulation of δ-subunit-containing extrasynaptic GABA-A receptors may lead to increased neuronal excitability and neurosteroid sensitivity, which could trigger postpartum excitability and PPD symptoms. Neurosteroid replacement therapy (NRT), involving neurosteroid administration during the perimenstrual or postpartum period, could manage these conditions without hormonal side effects. Based on the NRT platform, intravenous AP (renamed as brexanolone) was approved by the FDA to treat PPD, and oral neurosteroid analogs are currently in clinical testing

Neurosteroids have been investigated extensively for over 20 years to treat various neurological and psychiatric conditions (Reddy 2004, 2008, 2011, 2014; Younus and Reddy 2018). Neurosteroids ability to enhance protection against neuropsychological disorders by binding and activating most GABA-A receptor isoforms has been established (Reddy and Rogawski 2009, 2010, 2012). Neuroendocrine disorders, including catamenial epilepsy and PPD, are triggered by a sudden fluctuation in neurosteroids (Reddy 2009; Schiller et al. 2014). Neurosteroid replacement by administering exogenous neurosteroids is an effective therapy for neuroendocrine conditions associated with an abrupt fall or decline in AP. Neurosteroids, or their synthetic analogs, can be administered in a pulse protocol when endogenous levels abruptly fall (Fig. 3). A pulse protocol refers to cyclical administration of neurosteroids on specific days each month or during particular phases of the menstrual cycle, including the perimenstrual or periovulatory period. In catamenial epilepsy, low neurosteroid doses can be administered during the perimenstrual period or throughout the month to lessen seizure severity (Reddy 2016). Similarly, neurosteroids can be given for few days during the vulnerable period or administered daily for 2–3 weeks after the onset of PPD symptoms. Given the severity PPD and the lack of effective treatments, neurosteroid replacement strategy has been adopted as a therapy for PPD.

### Neurosteroid biosynthesis

AP is synthesized in the central nervous system (CNS) (Diviccaro et al. 2022) and like related neurosteroids, it is synthesized de novo in oligodendrocytes, astrocytes, Schwann cells, Purkinje cells, hippocampal neurons, retinal amacrine cells, and ganglion cells (Baulieu 1998; Melcangi et al. 2014; Pluchino et al. 2016, 2019). During pregnancy, neurosteroids are also synthesized in the placenta and corpus luteum (Rupprecht et al. 1996). Using circulating cholesterol or intermediate steroids, neurosteroid synthesis involves a series of A-ring reductions (Fig. 2) that is controlled by cytochrome P450, a cholesterol side-chain cleavage enzyme (CYP450scc). CYP450scc regulates steroid translocation into the mitochondria for initial biosynthesis and has been a focus of recent neurosteroid investigation (Rupprecht et al. 2010). A key enzyme in neurosteroid biosynthesis is 5α reductase, which can be inhibited by finasteride.

**Fig. 4:**
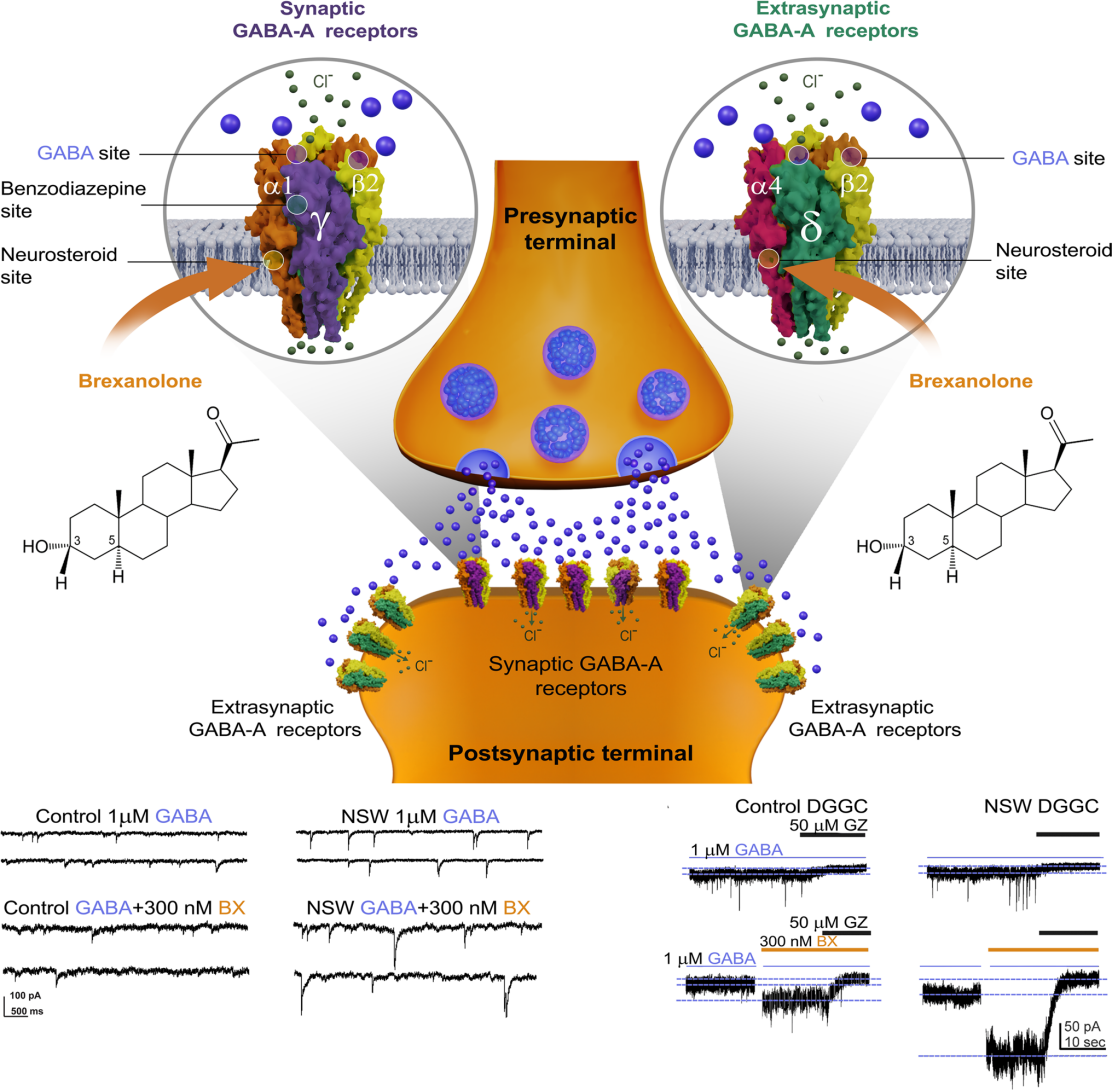
Schematic illustration of potential mechanisms of brexanolone action in the brain. Like other neurosteroids, BX binds to synaptic (γ2 subunit-containing) and extrasynaptic (δ subunit-containing) GABA-A receptors, including those that contain α4 and α6 subunits that are insensitive to benzodiazepines or those that lack the γ2 subunit necessary for benzodiazepine sensitivity. BX allosterically activates GABA-A receptor function, leading to hyperpolarization of neurons through an influx of chloride ions into the cell, a decrease in resting membrane potential, and hyperpolarization of the postsynaptic neuron and its network. By binding to and activating synaptic receptors, BX enhances phasic currents by increasing amplitude and decreasing decay of miniature inhibitory postsynaptic currents (mIPSC; left panel). BX binds to extrasynaptic receptors and enhances tonic current by a persistent surge in channel conductance without desensitization (right panel). This contributes to maximal network inhibition, a unique feature of the neurosteroid, but not benzodiazepines or other GABAergic drugs. Postpartum women have a markedly lower endogenous neurosteroids, referred as neurosteroid withdrawal, than the elevated levels experienced during pregnancy. However, the number of extrasynaptic GABA-A receptors may be elevated in the face of reduced AP levels. With administration of BX (injectable formulation of AP), the combination milieu rapidly replenishes allopregnanolone levels and expression of extrasynaptic GABA-A receptors is thought to contribute to rapid and effective relief of PPD symptoms due to inherent anxiolytic and anti-dysphoric effects of the neurosteroid. Thus, BX therapy aims to increase brain AP levels corresponding to the third-trimester pregnancy to mitigate PPD symptoms. GZ: gabazine; DGGC: dentate gyrus granule cells; NSW: neurosteroid withdrawal

Neurosteroids can be synthesized peripherally (often termed “neuroactive steroids”) and within the brain. Since their discovery in 1941, neurosteroids have been shown to have powerful neuro-modulatory effects in the brain (Selye 1941; Kulkarni and Reddy 1995). Their small structure and lipophilic properties allow them to cross the blood-brain barrier to exert their effects. The transport of cholesterol into the mitochondria is mediated by a translocator protein. By interacting with membrane-bound receptors, neurosteroids rapidly alter neuronal excitability by allosterically modulating GABA-A receptors (Carver and Reddy 2013; Chuang and Reddy 2018a) (Fig. 4), forming the basis for their therapeutic potential to treat epilepsy, acute and chronic pain, memory impairment, premenstrual dysphoric disorder, and PPD (Ratner et al. 2019).

### Neurosteroid interactions with GABA-A receptors

Over the past 20 years, the mechanisms of neurosteroid actions at GABA-A receptors have been clearly elucidated (Fig. 4). Structurally, GABA-A receptors are pentameric channels composed of various subunits, typically a combination of α1-6, β1-4, γ1-3, δ, ε, θ, ρ1-3, with the fifth subunit varying based on receptor type and location. Subunit composition determines the receptor’s sensitivity to drugs and other compounds, with δ-containing receptors being preferentially bound by neurosteroids. GABA-A receptors are classified as synaptic (primarily γ-containing) or extrasynaptic (primarily δ-containing) receptors based on their location on neuronal sites. Synaptic and extrasynaptic receptors differ in GABA affinity, desensitization rate, agonist efficacy, and neurosteroid sensitivity. Although neurosteroids can bind to all GABA-A receptor subtypes, they preferentially bind to δ-containing extrasynaptic receptors (Lan and Gee 1994; Reddy 2003b; Reddy 2010; Reddy and Estes 2016). Neurosteroids act as positive allosteric agonists of both synaptic and extrasynaptic GABA-A receptors. At high concentrations, neurosteroids directly activate receptors, while at low concentrations, they allosterically potentiate GABA-A receptor currents. Neurosteroids bind to specific “neurosteroid binding sites” on the receptor, which are distinct from binding sites for other drugs such as barbiturates or benzodiazepines. There are two discrete neurosteroid binding sites: one within the a-subunit transmembrane domain and another at the α-β subunit interface.

A recent study identified a consensus neurosteroid pharmacophore model for the activation of tonic currents at extrasynaptic δGABA-A receptors (Carver and Reddy 2016; Chuang and Reddy 2018). Patch-clamp studies using natural and synthetic neurosteroids revealed that alterations of the C17 or C20 neurosteroid molecule region significantly affected the activation of tonic currents. The C3α-OH group was crucial for functional activity of extrasynaptic receptors, as C3β-OH epimers were inactive. Of the analogs tested, AP and related pregnane analogs had the highest potency and efficacy in promoting tonic currents, while androstane analogs had the weakest response. The δ-subunit of GABA-A receptors was found to be essential for neurosteroid activity, as tonic current potentiation was significantly diminished in δ-knockout mice (Reddy 2018). These findings highlight the importance of δGABA-A receptors in neurosteroid sensitivity, tonic inhibition, and their therapeutic uses.

### Anxiolytic and antidepressant effects of neurosteroids

After isolating and characterizing neurosteroids, studies began to focus on their hypnotic, anxiolytic, and anti-seizure activities (Aird and Gordan 1951; Gyermek et al. 1967; Green et al. 1978; Reddy and Kulkarni 2000; Reddy 2010) (Table 2). Initial attempts to study neurosteroid anxiolytic activities showed that in stressful conditions, animals had decreased serum concentrations of AP (Lan and Gee 1994). Fluoxetine, an SSRI, was shown to increase local AP levels in the brain, but not in plasma, and AP activation was essential for fluoxetine’s anxiolytic and anti-dysphoric effects (Uzunova et al. 1996, 1998) (Table 3). Progesterone is a powerful anxiolytic and protective steroid (Reddy et al. 2004, 2005, 2010; Reddy and Apanites 2005). Moreover, anxiolytic responses induced by progesterone correlated significantly with increased AP levels in the blood and brain (Reddy and Kulkarni 1996, 1998). AP and related neurosteroids are potent anxiolytics with anti-dysphoric properties (Bitran et al. 1991; Reddy and Kulkarni 1997a, b, c; Reddy and Kulkarni 1998). Consistent with their GABAergic agonist properties, AP and related neurosteroids had anticonvulsant and anxiolytic effects in rodent models (Carver and Reddy 2013; Chuang and Reddy 2018a). Subsequently, AP and other GABAergic neurosteroids have been tested for protection against anxiety and seizures (Reddy and Kulkarni 1999; Reddy and Zeng 2007; Reddy, 2003). AP has shown protective effects in animal models of depression (Khisti et al. 2000; Evans et al. 2012; Almeida et al. 2018; Shirayama et al. 2020). Recently, ganaxolone, a synthetic analog of AP, was approved to treat seizures in CDKL5-deficiency epilepsy (Knight et al. 2022).

**Table 2:** Chronological preclinical studies by our team and others on neurosteroids and hormonal models relevant to postpartum depression

Finding	Reference
*Stage 1*: *Antidepressant and anxiolytic actions of neurosteroids*	
Anxiolytic activity of an endogenous adrenal steroid (1986)	Crawley et al. (1986)
Anxiolytic effects of allopregnanolone and progesterone (1991)	Bitran et al. (1991, 1995)
Role of GABA-A and mitochondrial DBI receptors in the antistress activity of neurosteroids (1996)	Reddy and Kulkarni (1996)
Differential anxiolytic effects of neurosteroids in the mirrored chamber behavior test (1997)	Reddy and Kulkarni (1997a)
Reversal of benzodiazepine inverse agonist FG 7142-induced anxiety syndrome by neurosteroids (1997)	Reddy and Kulkarni (1997b)
Neurosteroid coadministration prevents benzodiazepine withdrawal anxiety and hyperactivity (1997)	Reddy and Kulkarni (1997b)
The role of GABA-A and mitochondrial DBI receptors on the effects of neurosteroids on food intake (1998)	Reddy and Kulkarni (1998)
Antidepressant-like effects of neurosteroids in the depression test (1998)	Reddy et al. (1998)
Sex and estrous cycle-dependent changes in neurosteroid and benzodiazepine effects on behaviors (1999)	Reddy and Kulkarni (1999)
*Stage 2: Neurosteroid withdrawal models and therapeutic strategies*	
GABA-A receptor α4 subunit suppression prevents progesterone and neurosteroid withdrawal and resulting behavioral properties (1998)	Smith et al. (1998); Moran and Smith (1998)
NSW model of perimenstrual or postpartum-like excitability and seizures (2001)	Reddy et al. (2001)
Enhanced sensitivity of neurosteroid during perimenstrual NSW state (2001)	Reddy and Rogawski (2001)
Lack of tolerance to neurosteroids upon chronic administration (2000)	Reddy and Rogawski (2000a, 2000b)
Protective effects of stress-induced neurosteroids via GABA-ARs (2002)	Reddy and Rogawski (2002)
*Stage 3: Neurosteroid replacement therapy*	
Anti-seizure activity of progesterone and neurosteroids in PR-knockout mice (2004)	Reddy et al. (2004)
Anxiolytic activity of progesterone in PRKO model (2005)	Reddy et al. (2005)
Neurosteroid replacement therapy for catamenial epilepsy (2009)	Reddy and Rogawski (2009)
*Stage 4: NSW mechanistic rationale for neurosteroid-based therapies*	
GABA-A receptor plasticity during pregnancy and delivery (2008)	Maguire and Mody (2008)
Neurosteroid withdrawal regulates GABA-A receptor α4-subunit expression and seizure susceptibility (2010)	Gangisetty and Reddy et al. (2010)
Estrous cycle regulation of extrasynaptic receptors and tonic inhibition (2013)	Wu et al. (2013)
Identification of extrasynaptic mechanism for perimenstrual catamenial epilepsy (2014)	Carver et al. (2014)
PR-Independent regulation of GABA-A receptor plasticity (2017)	Reddy et al. (2017)
Catamenial-like seizure exacerbation in mice with targeted ablation of extrasynaptic δGABA-A receptors (2017)	Clossen and Reddy (2017)
Extrasynaptic receptor-mediated mechanisms in sex-differences in neurosteroids (2021)	Reddy et al. (2021)

**Table 3:** Preclinical studies of brexanolone (allopregnanolone) in anxiety and behavior models

Year	Reference	Objective	Key finding
1986	(Crawley et al. 1986)	Investigating THDOC’s anxiety-reducing properties in rats using the two-chambered test.	Moderate (10–15 mg/kg i.p.) doses of THDOC showed anxiolytic activities.
1991	(Bitran et al. 1991)	Investigating the anxiolytic effects of AP and its metabolites via i.c.v administration in plus maze test.	At highest administered dose (10 μm) animals showed both sedative and anxiolytic activity.
1994	(Lan and Gee 1994)	Measuring the correlation between fluctuating levels of AP and stress.	They reported that stress and the estrous cycle caused fluctuation levels of AP in the brain.
1995	(Bitran et al. 1995)	Progesterone effect on anxiety behavior.	Progesterone's anxiolytic effect was linked to increased AP levels.
1996	(Uzunova et al. 1996)	Assessing neurosteroid levels and behavior changes in animals after fluoxetine administration.	Fluoxetine increased AP, causing behavioral changes including anxiolysis.
1997	(Reddy and Kulkarni 1997a)	Animal behavior in mirrored chambers anxiety test following neurosteroid injection.	Neurosteroid AP reduces anxiety in animal models.
1998	(Pinna et al. 2003)	Effect of antidepressants on neurosteroid concentrations in animal models.	Lower neurosteroid levels were observed during periods of depression or social isolation.
2002	(Reddy and Rogawski 2002)	Investigating THDOC’s role in stress-induced seizures after acute stress in rats.	Stress-induced THDOC levels enhance seizure threshold and protection against excitability.
2003	(Reddy 2003a)	Investigating the physiological role of THDOC in stress.	THDOC mediated a positive reduction in stress.
2005	(Reddy et al. 2005)	Investigated the anxiolytic neurosteroids in PR-knockout model.	Demonstrated the anxiolytic effects independent of progesterone receptors.
2008	(Maguire and Mody 2008)	Investigated the postpartum behaviors in delta-KO mice	Mice with delta knockout exhibit abnormal postpartum behaviors
2018	(Melon et al. 2018)	Investigated the depression-like behaviors during postpartum period and deficits in maternal care in delta-KO models.	Treatment with neurosteroid SGE-516 reduced depression in postpartum delta-KO mice.

### Mechanisms of neurosteroid withdrawal, anxiety, and hyperexcitability

Fluctuations in neurosteroids affect anxiety and hyperexcitability. Neurosteroids are able to cause withdrawal in their absence, especially after prolonged exposure such as occurs during the menstrual cycle and pregnancy. Massive declines in neurosteroid expression, referred to as neurosteroid withdrawal (NSW), occur following the perimenstrual period (low progesterone state) and during the postpartum period after childbirth (low progesterone state). NSW is associated with increased neuronal excitability, anxiety, seizure susceptibility, and resistance to benzodiazepines and other anticonvulsants (Reddy and Rogawski 2001) (Gangisetty and Reddy 2010; Reddy 2016). Elevated progesterone during luteal phase and also during pregnancy serve as precursor for the biosynthesis of neurosteroids, specifically AP. NSW can be pharmacologically induced by finasteride, a 5α-reductase inhibitor that inhibits progesterone conversion into AP. Elevated levels of progesterone have anxiolytic and disease-modifying properties (Reddy et al. 2001; Reddy et al. 2010; Reddy et al. 2012; Reddy and Ramanathan 2012). These effects occur primarily due to its conversion into AP (Reddy et al. 2004, 2005). Animal models of NSW overexpress the GABA-A receptor α4-subunit (Smith et al. 1998) that is controlled by the Egr3 gene in a progesterone-independent pathway (Gangisetty and Reddy 2010). The increased expression of progesterone receptors can mediate the excitatory effects of progesterone when neurosteroid synthesis is blocked (Reddy and Mohan 2011). In addition, a catamenial-like seizure exacerbation is observed in kindled mice with targeted ablation of extrasynaptic δGABA-a receptors in the brain (Clossen and Reddy 2017). Furthermore, in the context of NSW, mice lacking the δ-subunit of GABA-A receptors exhibited diminished sensitivity to benzodiazepines. Interestingly, while wild-type animals demonstrated increased sensitivity to neurosteroids during NSW, δKO mice did not exhibit any changes in neurosteroid sensitivity. These findings suggest that alterations in the abundance of δGABA-A receptors contribute to increased catamenial seizure exacerbation and modifications in the responses to anti-seizure drugs, aligning with the effects induced by NSW. Thus, the NSW model has helped to confirm that traditional antiepileptics have a decreased ability to prevent catamenial seizures. Thus, NSW contributes to elevated anxiogenic excitability and seizure susceptibility in conditions associated with massive neurosteroid declines, as seen in PPD.

### Neurosteroid levels during pregnancy and the postpartum period

Reduced neurosteroid synthesis is a hallmark feature of PPD (Gilbert Evans et al. 2005; Meltzer-Brody and Kanes 2020). While some clinical findings are mixed, a prevalent hypothesis suggests that PPD symptoms may be attributed to the decline of progesterone-derived AP following childbirth, known as NSW as stead in the previous section (Reddy et al. 2001; Reddy 2016, 2022, 2023). During pregnancy, the concentration of AP and other neurosteroids increases, reaching their peak during the third trimester, then dropping sharply after delivery (Fig. 4). AP has been studied as a potential target molecule for PPD treatment because it is one of the most potent regulators of inhibitory GABA-A receptor function (Reddy 2022). Serum levels of AP in women during the follicular phase of the menstrual cycle are 0.8 nM (0.25 ng/mL) (Genazzani et al. 1998) and are highest during the third trimester of pregnancy, ranging from 26 to 70 nM; 8.15–22 ng/ml (Pennell et al. 2015; Gilbert Evans et al. 2005; Paoletti et al. 2006; Parizek et al. 2005) to 159 nM; ~50 ng/mL at parturition (Luisi et al. 2000; Hellgren et al., 2014). Consistent with these studies, significant changes in neurosteroid levels are reported in PPD. In studies by Deligiannidis et al. (2016), decreased GABA levels were linked to increased depression scores in women at risk of developing PPD. Other studies indicate that reduced AP levels are associated with increased depressive symptoms in pregnant women (Hellgren et al. 2014), while higher AP levels are associated with a lower risk of developing PPD (Osborne et al. 2017). Thus, an abrupt decline in neurosteroid levels and functional activity is a key pathology for depressive symptoms in PPD.

An alternate hypothesis for neurosteroid fluctuations in PPD is dysfunctional stress response (Maguire and Mody 2008). Clinical studies examining AP levels in PPD have yielded mixed results and often overlook the influence of stress on PPD. Interpreting perinatal studies on neurosteroid blood concentrations has proven challenging, with variations in methodology (RIA vs. LCMS) and the inclusion of women taking different psychotropic medications (Reddy 2023). Some studies demonstrate elevated AP concentrations in women with perinatal depression throughout pregnancy and postpartum, correlating with HAM-D scores and functional correlates. The concept that low AP characterizes PPD fails to acknowledge impaired stress reactivity and AP functioning in PPD. Hence, elevated serum AP in some PPD studies may serve as a marker of dysfunctional stress response during prolonged exposure to GABAergic neuroactive steroids (Meltzer-Brody and Kanes 2020). Moreover, reproductive mood disorders may be influenced more by sensitivity to changes in reproductive hormone levels than by absolute levels. While elevated AP levels, indicating a dysregulated stress response, have been identified as an additional marker of PPD, it is the sensitivity to changes in neurosteroids rather than absolute levels that likely plays a significant role in PPD (Carver et al. 2014; Reddy 2016). Traditional antidepressants are less effective for PPD due to the need for chronic dosing in serotonergic and noradrenergic pathways, but neurosteroid therapy shows promise in rapidly restoring network sensitivity by replenishing AP levels akin to those in the third trimester of pregnancy. Ongoing research on neurosteroids is helpful to elucidate the molecular pathophysiology of PPD.

## Neurosteroid replacement therapy (NRT)

Since PPD is characterized by reduced neurosteroid levels and increased depressive symptoms, neurosteroid mechanisms are providing new insights into the neuroendocrine basis of PPD and its potential amelioration (Fig. 2). Natural and synthetic neurosteroids have been investigated in a variety of models of anxiety, epilepsy, and related brain conditions (Reddy and Woodward 2004; Reddy 2009; Reddy 2013a; Reddy and Jian 2010; Chuang and Reddy 2019, 2020; Reddy et al. 2019, 2021). The NSW model has been used to develop new PPD therapies (Reddy 2022). While some anti-seizure medications (ASMs) can protect against perimenstrual catamenial seizures in women (a condition analogous to PPD), many women do not respond to common ASMs. In studies using the NSW model, conventional ASMs do not effectively protect against catamenial seizures. Surprisingly, neurosteroids and their synthetic analogs have been found to produce enhanced sensitivity to protect against seizures in the NSW-based perimenstrual model (Reddy and Rogawski 2000a, 2001). Unlike benzodiazepines, neurosteroids have enhanced sensitivity in NSW models (Reddy et al. 2010). In 2009, we proposed that NRT could effectively control the severity of catamenial seizures by administering a neurosteroid or synthetic analog in a pulse protocol during the perimenstrual period or throughout the month, at low doses to avoid side effects. The NRT strategy has been extended to PPD, which is associated with NSW and neurosteroid deficiency.

Preclinical research on AP and related neurosteroids has paved the way to identifying and developing NRT for PPD. Our team and others have extensively researched neurosteroid exposure and withdrawal models as well as their underlying pharmacological mechanisms (Table 2). For example, in the hippocampus, AP-potentiated GABA-gated currents can be blocked in a zinc concentration-dependent manner (Carver et al., 2016). Although its precise mechanism in humans is unclear, neurosteroid therapy involves a unique “extrasynaptic mechanism” that occurs during the perimenstrual and postpartum periods, offering a breakthrough PPD therapeutic strategy. Due to its potent effects on GABA-A receptor function, NRT has significant promise in treating neuroendocrine conditions, including PPD and premenstrual disorders (Reddy and Bakshi 2020). Although many neurosteroids are still in clinical investigations (Reddy and Estes 2016), BX has been FDA-approved to treat PPD. BX therapy for PPD is based on the core NRT, which provides postpartum mothers with AP doses equivalent to endogenous third-trimester concentrations for a few days (Meltzer-Brody and Kanes 2020). BX allosterically modulates and directly activates extrasynaptic δGABA-A receptors in the dentate gyrus, a brain region associated with memory, epilepsy, and psychiatric disorders (Carver et al. 2014). The tonic inhibition produced by BX depends on the δ-subunit and is mediated by protein kinase C (PKC) activity (Chuang and Reddy 2018). Our previous work on BX and NSW models led to this innovative NRT therapeutic strategy and eventually to PPD treatments (Table 2).

## Development of brexanolone for PPD

Brexanolone (BX) is an aqueous formulation of the neurosteroid AP (Table 3). The lipophilic BX molecule readily passes through the blood-brain barrier, allowing it to exert effects in the brain. The direct interaction of BX with GABA-A receptors can produce anesthetic, anticonvulsant, antidepressant, and anxiolytic effects (Reddy and Kulkarni 1996; Reddy and Kulkarni 1997a; Reddy and Kulkarni 1997b; Reddy and Kulkarni 1998; Reddy and Kulkarni 1999; Reddy et al. 1998; Reddy and Rogawski 2000a; Reddy et al. 2004, 2005; Clossen and Reddy 2017). BX can be reversibly converted into 5α-dihydroprogesterone, a steroid hormone with a high affinity for progesterone receptors (Rupprecht et al. 1996). The steroid 5α-dihydroprogesterone has a key role in reproductive function and seizure susceptibility (Reddy and Mohan 2011). Thus, BX has gained interest as a treatment modality for neuroendocrine conditions. The precise mechanism by which BX inhibits PPD is unclear; however, its therapeutic effects are thought to occur by activation of synaptic and extrasynaptic GABA-A receptors. Current guidelines require intravenous (IV) administration, as BX has less than 5% bioavailability when given orally (Kanes et al. 2017a). With IV administration, FDA-approved BX has a plasma clearance rate of 0.8 L/h/kg, with a half-life of 40 min. To maintain adequate plasma concentrations for effective therapeutic benefit, BX requires a continuous 60-h infusion (Frieder et al. 2019). The optimal pattern of BX administration to maintain effective levels and cause PPD remission is 30 μg/kg/h during the first 4 h, followed by an increase to 60 μg/kg/h over the next 20 h, and another increase to 90 μg/kg/h until hour 52. At this point, a reduced dose of 60 μg/kg/h can be used if 90 μg/kg/h is not well tolerated. Hours 52 to 60 are considered tapering hours, with a reduced dose of 60 μgkg/h in hours 52 to 56 and an additional reduction to 30 μg/kg/h in the last 4 h.

## Preclinical profile of brexanolone 

Unlike reproductive steroids, neurosteroids do not cause hormonal activity but do have rapid nervous system effects, including hypnotic, anxiolytic, and anticonvulsant actions (Kulkarni and Reddy 1995; Reddy 2010). In 1986, the adrenal-derived neurosteroid allotrahydro-deoxycorticosterone (THDOC) was shown to interact with the GABA-A receptor in a manner similar to barbiturates, allowing benzodiazepines, leading to faster receptor binding (Crawley et al. 1986). This activity prompted researchers to analyze THDOC more thoroughly, as its’ behavioral effects are similar to those of established anxiolytics (Reddy and Rogawski 2002). The chronological studies of BX and related neurosteroids by our team are listed in Table 2 and the preclinical anxiolytic profile of BX is outlined in Table 3. Initial attempts to study the anxiolytic effects of BX showed that animals in stressful environments had lower serum AP levels (Evans et al. 2012; Reddy et al. 2022). This observation was originally thought to be a result of stress, as opposed to its cause, but decades of neurosteroid research proved this to be untrue (Meltzer-Brody 2011; Sriraman et al. 2015; Cox et al. 2016;). Bitran and team reported anxiolytic effects after treating ovariectomized rats with progesterone and AP (Bitran et al. 1991, 1995). While studying the mechanism by which progesterone alters behavioral anxiety phenotypes, they found that progesterone-induced anxiolytic responses correlated highly with increased AP levels in the blood and brain. It was thought that a reduction in the amount of AP is the cause of excessive anxiety and excitability. However, the precise mechanism remained unexplored for years.

In 1996, fluoxetine was shown to increase local AP levels in the brain but not in plasma (Uzunova et al. 1996). Anxiolytic effects were observed in animals treated with fluoxetine, and while researchers did not credit these observations to increased AP levels, they concluded that AP activation could be critical to the anxiolytic and anti-dysphoric effects of fluoxetine. In 1997, we evaluated the differential anxiolytic effect of neurosteroids (Reddy and Kulkarni 1996; Reddy and Kulkarni 1997b; Reddy and Kulkarni 1998) using a mirrored chamber test of anxiety following injections of AP, progesterone, and 4′-chlordiazepam, a specific ligand for the mitochondrial diazepam-binding inhibitor receptor. A clear and robust dose-dependent anxiolytic response was seen in animals treated with progesterone or AP. The anxiolytic effects of progesterone occur when it is converted to neurosteroids in the brain (Reddy et al. 2005). Extensive studies conducted by our lab and others on psychopharmacological effects of neurosteroids (Table 2) (Reddy and Kulkarni 2000) have paved the way for further research on the pharmaceutical potential of neurosteroids to treat depressive conditions.

## Clinical profile of brexanolone

BX is the first drug approved for clinical use in PPD. AP’s role in postpartum blues was first reported in 2001 (Nappi et al. 2001). Serum AP levels were significantly lower in women with postpartum blues than in those who did not meet PPD criteria. Progesterone levels, however, did not differ significantly between the two groups. These results provided evidence that maintaining stable AP levels in postpartum women was important. We and others have shown that neurosteroids play a key role in regulating menstrual conditions, which promoted the development of NRT, i.e., administering exogenous neurosteroids to levels that activate GABA-A receptors (Reddy 2016, 2022). These studies ultimately led to clinical development of BX. In 2014, clinical trials (four studies: two in phase II and two in Phase III, outlined below) were initiated to evaluate the potential use of BX in PPD (see Table 4). These studies showed a clinically beneficial effect of BX, with improvement in depressive symptoms on the Hamilton Depression Rating Scale (HAM-D) that is consistent with other antidepressants and faster than other treatments (60 h versus 4 weeks). The HAM-D remission criteria, which require a total score of < 10 and > 50% reduction, also support the efficacy of BX. Additionally, the Clinical Global Impression of Improvement (CGI-I) showed statistically significant and clinically meaningful differences in BX from the placebo. While few other endpoints were statistically significant, they all had fewer depressive symptoms with BX, including several patient-rated scales, including the Barkin Index of Maternal Functioning and Edinburgh Postnatal Depression Scale. These clinical studies of BX are critical to its FDA approval (Table 5).

**Table 4:** Summary of brexanolone clinical trials in postpartum depression

	Study (sample size)	Study design	Intervention and comparator	Primary objective (time period)	PPD diagnosis criteria (timing of onset)	Key efficacy outcome	References
Phase II	(Pilot study) (*N* = 4)	Open-label, proof of concept	Subjects received BX (90 μg/kg/hr)	To assess change in HAM-D-17, EPDS, GAD-7 and PHQ-9 scores (at 60- and 84-hour post administration)	HAM-D-17 score ≥ 20 (postpartum)	Significant reduction in HAM-D, EPDS, GAD-7, and PHQ-9 scores after infusion. HAM-D total score decreased 81% from baseline at Hour 84	(Kanes et al. 2017a)
	202A (*N* = 21)	Double-blind, randomized, placebo-controlled	1:1 BX90 group (*N* = 10) and placebo group (*N* = 11)	To assess the change in HAM-D-17 score from baseline. (60 hours)	SCID-I MDE and HAM-D-17 ≥ 20 (third trimester to 4 weeks after birth)	BX90 group showed significant reduction in HAM-D score (−21 points) compared to placebo group (+8.8 points) at 60 hours	(Kanes et al. 2017b)
Phase III	202B (*N* = 108)	Double-blind, randomized, placebo-controlled	1:1:1 BX90 (*n* = 45), BX60 (*n* = 47), or placebo (*n* = 46)	To assess the change from baseline in the 17-item HAM-D total score (60 hours)	HAM-D score ≥ 26	BX60 and BX90 groups showed greater reduction in HAM-D score (−19.5 and −17.7 points, respectively) than placebo (−14 points) at 60 hours	(Meltzer-Brody et al. 2018b; Meltzer-Brody et al. 2018a)
	202C (*N* = 108)	Double-blind, randomized, placebo-controlled	1:1 BX90 (*n* = 54) or placebo (*n* = 54)	To assess the change from baseline in the 17-item HAM-D total score (60 hours)	HAM-D score of 20–25	BX90 group showed greater reduction in HAM-D score (−14.6 points) compared to placebo (−12.1 points) at 60 hours.	NCT03665038, 2018; Meltzer-Brody et al. 2018a)
	BX for adolescent (*N* = 28)	Double-blind randomized and open-label	1:1:1.5 BX90 (*n* = 8) Placebo (*n* = 8) Open-label BX (*n* = 12)	To evaluate safety and tolerability of BX in adolescents (age between 15 and 17) suffering from postpartum	PPD diagnosed by DSM-5 axis I Disorder (SCID-5)	Preliminary data suggests similar effectiveness of BX and placebo in adolescents, with 50% efficacy for placebo and 37.5% for BX in double-blind study and 41.7% remission with BX in open-label study	(History of Changes for Study: NCT03665038, 2022)

**Table 5:** Overview of brexanolone for PPD

*Brexanolone (allopregnanolone) IV injection FDA approval: 2019*	
*Clinical indications:* For the treatment of PPD in adult women (18 to 45 years of age) *Product and strengths:* Approved indications: postpartum depression in adult women Product strengths: 5 mg/ ml solution in single dose vials (100 ml) Formulation: admixed in 25% β-sulfobutyl-ether sodium solution Expiry period: 36 months (stored in refrigerator) Storage: diluted solutions can be stored for a maximum of 12 hours only *Recommended dosage:* Continuous IV infusion over a total of 60 hr (2.5 days). Recommended infusion rate as follows: 0 to 4 hours: start with a dose of 30 μg/kg/hour 4 to 24 hours: increase the dose to 60 μg/kg/hour 24 to 52 hours: increase the dose to 90 μg/kg/hour 52 to 56 hours: decrease the dose to 60 μg/kg/hour 56 to 60 hours: decrease the dose to 30 μg/kg/hour *Clinical pharmacokinetics:* Oral bioavailability: < 5% Volume of distribution (*V*_d_): 3 L/kg Metabolism: non-CYP pathways; its main metabolism routes include ketoreduction, glucuronidation, and sulfation pathways. Half-life (*t*_1/2_): 40 minutes Effective half-life: 9 hours Clearance (CL): 0.8 L/h/kg Protein binding: 99% *Long-term safety:* BX is approved for one treatment per postpartum period and not indicated for continuation of therapy. Long-term efficacy data after a 30-day follow-up period is not available. Thus, the durability of treatment effect beyond 30 days is unknown. *Adverse effects:* common adverse effects are dizziness, sedation/somnolence, xerostomia, loss of consciousness, and hot flashes. *Drug interactions:* due to its metabolism by multiple enzymes, it is unlikely for BX to be a substrate of metabolic interactions with a concomitant drug. Metabolized by extra-hepatic pathways; no dosage adjustment is necessary with hepatic impairment. Concomitant use of BX with other CNS depressants (benzodiazepines, opioids) may increase severity of adverse reactions related to sedation. *Warning and precautions:* should not be used in patients who have a glomerular filtration rate of < 15 mL/minute/1.73 m^2^ (potential renal effect of cyclodextrin solution). *Black box warnings:* excessive sedation and sudden loss of consciousness. Due to the risk of serious harm in patients treated with brexanolone, monitoring for excessive sedation and sudden loss of consciousness and continuous pulse oximetry is required. Patients must be accompanied during interactions with their child. *Risk evaluation and mitigation strategy (REMS*)*:* due to the risk of excessive sedation or sudden loss of consciousness with BX, the drug is administered only to patients in a medically supervised setting that provides monitoring during administration; pharmacy and healthcare facilities should be certified and must be enrolled in REMS program. To meet REMS requirements, a provider must be on-site for the entire 60-hour treatment to monitor the patient (and intervene, if necessary). Monitoring must include pulse oximetry and evaluation for excessive sedation every 2 hours during planned periods. *Controlled substance:* brexanolone is a Schedule IV controlled substance as per the DEA.	

### Phase 2 clinical trials

The first phase II randomized controlled trial (201A) conducted by SAGE evaluated the safety and efficacy of BX using the HAM-D score (Kanes et al. 2017a). Women with a history of seizures, active psychosis, or significant organ disease were excluded from participation. Researchers enrolled four patients aged 18 to 45 that suffered major depressive episodes within 4 weeks of giving birth. BX administration began with constant IV infusion for 36 h, followed by a 12-h tapering period. By the last evaluation, HAM-D scores were found to be similar to those in PPD remission. Fourteen mild adverse events were recorded with no fatalities. This study design provided a strong foundation for subsequent trials.

Two years later, the second phase II trial (202A) was conducted using double-blind, placebo-controlled design (Kanes et al. 2017b). In this trial, 21 women aged 18 to 45 were recruited using the same exclusion criteria as 201A. Participants in this trial had severe PPD, reflected by a HAM-D score ≥ 26. Qualified participants were randomized 1:1 into either an experimental group that received a 60-h BX infusion (*n* = 10) or a placebo group (*n* = 11). Both groups were infused at a rate of 90 μg/kg per hour for 28 h, followed by a tapering dose of 60 μg/kg per hour for 4 h, and 30 μg/kg per hour for the last 4 h. The efficacy of BX was evaluated based on a reduction in HAM-D score. At 60 h, a 21-point mean reduction from baseline was found in total HAM-D scores among the BX group, compared to an 8.8-point reduction for those in the placebo group. Thus, BX infusion produced a significant and clinically meaningful reduction in HAM-D total score. Adverse events were recorded in 4 of 10 patients in the BX group, including symptoms of dizziness and somnolence, versus 8 of 11 patients in the placebo group. This study demonstrated the efficacy of BX in PPD remission, which led to phase III trials.

### Phase 3 clinical trials

The pivotal phase III consisted of two multicenter, double-blinded, randomized, placebo-controlled trials (Meltzer-Brody et al. 2018a). These studies recruited a total of 375 postpartum participants aged 18 to 45 with severe depression. Similar to phase II, participants were required to have ceased breastfeeding before study initiation. History of active psychosis, attempted suicide, bipolar disorder, schizophrenia, and/or schizoaffective disorders disqualified participants from recruitment. Other exclusion criteria included a history of renal failure that required dialysis, anemia, and known allergies to BX or progesterone.

The first trial (202B) recruited women with HAM-D scores of 20 or higher (Meltzer-Brody et al. 2018b). Participants were randomly assigned 1:1:1 to receive group 1: BX at 90 μg/kg per h (BX90), *n* = 45; group 2: BX 60 μg/kg per h (BX60), *n* = 47; or group 3: a matched placebo, *n* = 46. Participants received a dose of 30 μg/kg/h during hours 0 to 4 of infusion, 60 μg/kg/h during hours 4 to 24, and 90 μg/kg/h during hours 24 to 52. The dose was tapered down to 60 μg/kg/h during hours 52 to 56, and to 30 μg/kg/h during the final 4 h. At 60 h, the mean reduction HAM-D total score was 19.5 points in group 1 (*p* = 0.0013), 17.7 points in group 2 (*p* = 0.0252), and 14 points in the placebo group 3. In group 2 (60 μg/kg), 19 participants experienced adverse events, while 22 participants in groups 1 (90 μg/kg) and 3 (placebo) experienced adverse events. Common adverse effects included headaches, dizziness, and somnolence. The second phase III trial (202C) recruited postpartum women with HAM-D scores of 25 or higher (Meltzer-Brody et al. 2018a). One hundred eight participants were randomly assigned 1:1 to receive BX at 90 μg/kg per h (*n* = 54) or placebo (*n* = 54). The 60-h BX infusion was administered in the same pattern as 202B. At 60 h, the mean reduction in HAM-D score was 14.6 in the BX90 group and 12.1 in the placebo group (*p* = 0.0160). Headache, dizziness, and somnolence were the most frequently reported side effects. One patient experienced altered consciousness and syncope, which was determined to be related to BX therapy. Overall, BX injection produced significant and clinically meaningful reductions in the HAM-D total score at 60 h compared with placebo, with rapid onset of action and effective response during the study period. These trials were pivotal studies for FDA approval of BX for PPD, and this therapy is currently available at certified health facilities with active monitoring by healthcare providers.

## Safety profile of brexanolone

BX is proven to be safe for use in PPD but has poor oral bioavailability (< 5%); hence, it is administered by continuous IV infusion (Table 5) (Gunduz-Bruce et al. 2022). Its distribution volume is 3 L/kg, suggesting extensive distribution into tissue. About 99% of BX binds to proteins, independent of its plasma concentration. About 47% of BX is eliminated in feces, and 42% in urine. In humans, BX is metabolized by non-CYP pathways. BX is primarily metabolized by ketoreduction, glucuronidation, and sulfation pathways. The resulting metabolites are sulfate or glucuronide conjugates of C20-reduced forms of BX. Because BX is metabolized by multiple enzymes, it is not likely to be a substrate of metabolic interactions with a concomitant drug. However, drug interactions have not been widely tested in combination with BX.

### Side effects and serious adverse events

Side effects and serious adverse events were noted with BX. In clinical trials, somnolence, headaches, dry mouth, and sedation were the most commonly observed side effects. Other adverse reactions included suicidal ideation, dizziness, presyncope, vertigo, tachycardia, and hot flashes. One subject experienced severe somnolence that caused less than 1 min of apnea during drug administration. This event required intervention to interrupt dose administration for 15 min to 1 h.

BX is indicated for the management of PPD (Meltzer-Brody and Kanes 2020), but its use requires patients to participate in a risk evaluation and mitigation strategies (REMS) program. Because BX infusion has been associated with loss of consciousness in some patients, continuous vigilance throughout the duration of treatment is required. Despite promising clinical evidence, a REMS program requirement and the tedious logistics of prolonged infusions are major treatment barriers for many deserving patients.

### Drug interactions

BX is not directly affected by metabolic interactions with other drugs due to its metabolism by multiple hepatic enzymes. Hepatic impairment does not require dosage adjustment since BX is metabolized through extra-hepatic pathways. However, caution must be exercised when using BX concurrent with benzodiazepines or opioids, as it may increase sedation and the severity of adverse effects. BX carries a Black box warning due to the risk of excessive sedation and sudden loss of consciousness. To address this issue, a REMS program was implemented. Pharmacological drug interaction studies identified a potential therapeutic interaction between BX and zinc and animal studies indicated that elevated zinc levels in the brain can diminish the protective effects of BX when administered simultaneously with zinc.

### Potential risk in special population

BX has not been thoroughly tested in special populations, including young adults, pre-partum, and breastfeeding patients, as well as those with concurrent hepatic and/or renal dysfunction. The BX injection solution is formulated in citrate buffered sulfobutylether-β-cyclodextrin (SBECD), which can accumulate in patients with severe renal impairment (Luke et al. 2010). Thus, BX use should be avoided in patients with end-stage renal disease or an estimated glomerular filtration rate (eGFR) of < 15 mL/min/1.73 m^2^ due to toxic accumulation of the SBECD solubilizing agent used to formulate the BX solution. In patients with severe renal impairment (eGFR 15–29 mL/min/1.73 m^2^), SBECD AUC_inf_ increased 5.5-fold and Cmax increased 1.7-fold. Thus, BX is contraindicated in patients with end-stage renal disease.

### Potential risk on infant brain development

Limited data on the effects of BX on infant brain development is available. In neonatal rat preclinical studies, administering BX caused widespread apoptotic neurodegeneration in the developing brain (Olney et al. 2002; Tian et al. 2020). A 2019 FDA report from animal studies of drugs that enhance GABAergic inhibition suggested that BX may cause fetal harm. No data on BX use in pregnant women is available to determine drug-induced risks of major birth defects or adverse maternal or fetal outcomes. Animal reproductive studies identified serious adverse outcomes. Salient findings in animal studies include (i) pregnant rats treated with 5 times the recommended BX dose displayed maternal toxicity, decreased pup viability, and neurobehavioral deficits in offspring; (ii) administering drugs that enhance GABAergic inhibition to neonatal rats can lead to widespread apoptotic neurodegeneration in the developing brain; and (iii) the vulnerable period for these changes in rats (postnatal days 0–14) corresponds to the third trimester of pregnancy in humans, which is crucial for brain development.

### Pregnancy and lactation

The FDA report provides evidence of risks associated with BX use during pregnancy and lactation. In studies of pregnant rats and rabbits, high BX doses resulted in decreased fetal body weight, increased resorption, and post-implantation loss in rabbits. When administered throughout pregnancy and lactation, higher doses led to higher pup mortality and reduced live births. Additionally, female offspring exposed to high BX doses had a neurobehavioral deficit. Lower doses did not show significant effects. Although the estimated background risk of major birth defects and miscarriage in the PPD population is unknown, all pregnancies carry a background risk of birth defects, loss, or other adverse outcomes. In the general population of the USA, the estimated background risk of major birth defects in clinically recognized pregnancies is 2 to 4%, while the risk of miscarriage is 15 to 20%. There is a registry to track pregnancy outcomes in women who have been exposed to antidepressants during pregnancy and physicians are encouraged to register eligible patients to the National Pregnancy Registry for Antidepressants.

The potential effects of BX on the neonatal brain are a concern because BX can be transferred to breastmilk in nursing mothers. BX levels in breastmilk were < 10 ng/ml, which is 1.37 times the plasma concentration in adult lactating women treated with IV BX according to the 60-h dosing regimen. Because BX has a bioavailability value of < 5% when administered orally, the relative dose administered to infants through breastmilk is estimated to be low. BX concentrations were reported recently in break milk and plasma in healthy volunteers given BX injection (Wald et al. 2022). BX time-course profiles were similar between breast milk and plasma. Given the rapid elimination of BX, the estimated relative infant dose was low. Whether BX affects breastmilk production is unknown. Transient somnolence is a potential risk for breast-fed infants of mothers receiving BX, but the likelihood of this occurring is low due to the relative infant dose and bioavailability. Regarding lactation, guidelines for breastfeeding women from the American College of Obstetricians and Gynecologists (ACOG Committee on Practice Bulletins--Obstetrics 2008) state that the benefits of breastfeeding must be carefully considered relative to potential risks to the baby from exposure to medications in breast milk. Some antidepressants are deemed safer for breastfeeding women than others, although long-term effects on exposed infants are uncertain. These and other recommendations apply to traditional antidepressants but not specifically to neurosteroids (Weissman et al. 2004; Academy of Breastfeeding Medicine Protocol Committee 2008). Because there may be a potential risk with BX, it is important to consider ways to decrease infant exposure to the medication. Some physicians recommend mothers stop lactating or if actively breastfeeding, consider ceasing breastfeeding at the start of BX infusion and for 4 days after the infusion ends.

### Risk of suicidal thoughts and behaviors

BX is approved for adult women; its effects on postpartum mothers under 18 years of age have not been evaluated (Gunduz-Bruce et al. 2022), nor has the safety and efficacy of BX in geriatric patients been established. Antidepressants that target the monoaminergic system have risks of suicidal thoughts and behaviors in patients aged 24 years and younger. BX has not been evaluated for these risks and directly targets the GABAergic system thus, the risks of developing suicidal thoughts in this age group must be determined. Concomitant use of BX with other CNS depressants (benzodiazepines, opioids) must also be studied as they may synergistically interact and increase the severity of adverse reactions related to sedation.

## Meta-analysis of brexanolone efficacy

After approval of BX as a PPD treatment, several meta-analyses were conducted to evaluate its safety, tolerability, and efficacy (Table 6). The first study, published in 2019, used data from 202A, 202B, and 202C (Zheng et al. 2019). Their results were analogous to the clinical trial data, concluding that BX resulted in significantly more PPD remission (RR = 1.86, 95% CI = 1.03–3.34). The second meta-analysis compared efficacy data between BX and SSRIs (Cooper et al. 2019). Six studies were selected from English databases for indirect treatment comparison. Researchers conducted a matching-adjusted indirect comparison and recorded changes from baseline HAM-D scores at time points ranging from administering the drug to initial signs of remission. Patients treated with BX had a larger change from baseline in HAM-D scores at all time points than those treated with SSRIs. Researchers acknowledged the weaknesses of indirect comparison but concluded that BX was superior to SSRIs in the treatment of PPD. In the most recent meta-analysis (Vatturi et al. 2020), researchers pooled data from 247 patients across three studies and calculated changes in HAM-D scores from baseline. Compared to placebo, BX infusion was associated with a mean reduction of 2.2 points from baseline. These meta-analyses increased the generalizability of results from individual trials, providing further evidence of the benefit of BX use in PPD treatment.

**Table 6:** Meta-analysis studies of brexanolone efficacy in postpartum depression

Study	Zheng et al. (2000)	Cooper et al. (2019)	Vatturi et al. (2020)
Year published	September 2019	October 2019	May 2020
Aim of the study	BX’s safety, tolerability, and efficacy in PPD compared to placebo	Comparison between BX efficacy with those of SSRIs	Evaluate the efficacy and safety of BX for PPD treatment
Data bases used	Chinese and American databases	English databases	Medline, Embase, and PsycINFO databases
# of studies used	3 studies	6 studies	3 studies
Studies used	Clinical trial studies (202A, 202B, and 202C)	Appleby et al. 1997; Kanes et al. 2017a; Meltzer-Brody et al. 2018a; Misri et al., 2004; O’Hara and Engeldinger 2018; Sharp et al. 2010; Yonkers et al. 2001	Clinical trial studies (202A, 202B, and 202C)
Unit of measure	Change in HAM-D score	Used ITC and matching-adjusted indirect comparisons (MAICs) to compare change in HAM-D scores	Change in HAM-D scores
Findings	BX significantly improved PPD remission rates with an RR of 1.86 and a 95% CI of 1.03 to 3.34 compared to placebo.	MAICs demonstrated greater improvement from baseline with BX compared to SSRIs. The differences between BX and SSRIs on the HAM-D were 12.79 at day 3, 5.87 at week 4, and 0.97 at the final observation.	BX infusion was associated with a mean reduction of 2.2 (95% CI: −3.6, 8.0) in HAM-D from baseline.

## Limitations of brexanolone

BX is the first drug therapy approved by the FDA for PPD in women (18 to 45 years of age) experiencing depressive symptoms during pregnancy or up to 4 weeks after delivery. BX is available as a 5 mg/mL solution in the β-cyclodexrin SBECD, which is administered as an intravenous infusion over 60 h. Once mixed, the solution is only stable for 12 h at room temperature and 96 h if refrigerated and the BX injection vial has a 36-month expiry period (refrigerated storage). The efficacy of BX suggests the importance of neurosteroids in treating PPD. However, certain issues with BX limit its widespread clinical use in PPD. Namely, it is unclear if BX provides long-term relief of depressive symptoms beyond 30 days after administration, and there is no data on long-term efficacy or potential relapse.

Clinical access to BX therapy has some barriers. BX is prescribed by, or in consultation with, a board-certified psychiatrist and obstetrics and gynecology physicians who met REMS program requirements in a healthcare facility certified by the REMS program. Currently, the only available administration method is a 60-h continuous infusion that requires inpatient hospitalization. This creates disparity for patients of lower socioeconomic status, as it requires BX recipients to cover hospitalization expenses, as well as high treatment costs (Eldar-Lissai et al. 2020). Moreover, patients receiving BX infusion require inpatient hospitalization for multiple days, which decreases treatment access for mothers with limited social support or limited health insurance. Alternative antidepressant therapies take weeks to months to reach full effect and often require chronic dosing to achieve a desired response. These issues can be alleviated by orally administered neurosteroids. However, the BX REMS program, which was adopted to limit severe adverse effects of administering BX, also limits drug availability to patients in need of emergent, rapid treatment.

Other limitations of BX include its efficacy to treat PPD, which is currently limited to 30-day observation. Thus, the long-term benefits of BX, and whether it can relieve depressive symptoms beyond 30 days following injection, need to be determined (Cornett et al. 2021). A synthetic neurosteroid, zuranolone, has been tested in patients with major depression (Clayton et al. 2023), with rapid alleviation of depressive symptoms using 30 mg oral zuranolone at days 3, 8, and 12. However, its overall benefit did not meet the primary endpoint, and ultimately, there was no evidence of zuranolone efficacy beyond 2 weeks.

BX and related neurosteroids have other biopharmaceutical limitations including lack of water-solubility, poor bioavailability, short half-life, and rapid hepatic inactivation, necessitating frequent administration or continuous infusion (Reddy and Rogawski 2012). Elevated zinc levels also diminish the protective effects of BX (Carver et al. 2016). These issues pose great hurdles for the therapeutic application of BX. There is an unmet medical need to engineer novel synthetic neurosteroids that overcome these limitations. Aqueous solubility is a key feature of injectable neurosteroid formulations for PPD therapy. Orally active neurosteroids offer greater access and improved drug delivery for affordable treatment. To overcome these issues, recent studies have focused on creating new analogs with improved potency and efficacy, considering blood-brain barrier permeability, aqueous solubility, oral absorption, and slower metabolism for longer half-lives (Carver and Reddy 2016; Zorumski et al. 2019; Althaus et al. 2020). Orally active neurosteroids with GABA-A receptor-modulating activity have been developed and tested in clinical trials. Zuranolone or SAGE-217 (30 mg once daily for 2 weeks) has been tested in women with PPD, showing significantly decreased depressive symptoms as early as day 3 that were sustained through day 45 (Deligiannidis et al. 2016). Phase 3 trials in adults with major depressive disorder (Clayton et al. 2023) found that oral zuranolone (20 and 30 mg) is well tolerated in patients with major depressive disorder, but it failed to significantly improve depression symptoms during chronic treatment (≥ 15 days). The findings suggest that while neurosteroids demonstrate selectivity in alleviating symptoms of PPD, they do not provide long-term relief in major depression. Benzodiazepines, which enhance synaptic GABA-A receptors but not extrasynaptic receptors (Reddy et al. 2015), are not effective in treating PPD or major depression. These findings collectively highlight the importance of targeting extrasynaptic GABA-ARs as significant therapeutic targets for PPD.

We recently developed cost-effective ways to administer neurosteroids with better efficiency and safety (Chuang and Reddy 2018) by developing novel water-soluble neurosteroids with improved biopharmaceutical properties (Reddy 2023). Valaxanolone and lysaxanolone are identified as lead hydrophilic synthetic neurosteroids with desirable biopharmaceutical profiles. These synthetic analogs were engineered to improve options for developing injectable and oral products. They are prepared as dry powder to be mixed with aqueous buffer prior to injection. Whereas BX requires complex formulations with β-cyclodextrins that have potential side effects and limits on the daily administration, water-soluble BX analogs provide better options with more stability and improved drug delivery (Reddy 2023). These analogs also have the potential to overcome issues including bioavailability, rapid metabolism, and extrasynaptic receptor selectivity as per the pharmacophore model (Carver and Reddy 2016). The most promising drug-like BX analogs are being selected for advanced development and clinical use in PPD and seizure disorders.

## Conclusions and future perspectives

PPD is a major depressive episode that occurs during pregnancy or within 4 weeks of delivery. As a highly prevalent, underdiagnosed mood disorder in postpartum women, PPD is characterized by emotional, cognitive, and behavioral disturbances, sharing symptoms with other forms of depression, such as sadness, anhedonia, cognitive impairment, and suicidal ideation. Due to the risk of suicide and negative effects on maternal-infant bonding and infant development, PPD is a life-threatening condition. Until the approval of BX, standard treatments for PPD were psychotherapy or traditional antidepressants, which often took several weeks to show efficacy. No specific PPD therapy existed until 2019, when neurosteroid research achieved a major PPD milestone. Neurosteroids are powerful anxiolytic and antidepressant compounds due to their rapid activation of synaptic and extrasynaptic GABA-A receptors in the brain. This has been demonstrated in preclinical studies including neurosteroid withdrawal models. Consequently, NRT was introduced to replenish adequate neurosteroid levels in situations of neurosteroid withdrawal or deficiency, such as PPD. Clinical trial data has shown the ability of BX to alleviate PPD symptoms within hours of initial administration (Edinoff et al. 2021; Gunduz-Bruce et al. 2022). This timeline for symptom relief is a vast improvement over that of antidepressants such as SSRIs and SNRIs. In contrast to complex mechanisms of traditional antidepressants, neurosteroids offer an improved approach to PPD treatment with advantages of known mechanism, rapid symptom relief, and persistent tonic inhibition.

Based on extensive preclinical research on neurosteroids and the NRT concept, BX, an injectable version of the endogenous neurosteroid AP, was developed to specifically treat PPD. It was the first drug to receive FDA approval to treat PPD. Two pivotal clinical trials showed that BX is effective with minimal drug interactions and few side effects (Gunduz-Bruce et al. 2022). In these studies, BX significantly improved depressive symptoms on the HAM-D within 60 h, which is much quicker than other available treatments that typically take 4 weeks (Epperson et al. 2023). HAM-D remission criteria were also met, and the CGI-I differed substantially from the placebo, indicating that BX can reduce depressive symptoms. The most commonly reported side effects of BX are headache, dizziness, and drowsiness. However, in rare cases, patients experienced suicidal thoughts, changes in consciousness, and fainting, so medical supervision is recommended when taking BX. Based on neuroendocrine insights, BX is rationale drug that addresses two significant pathologies related to PPD: neurosteroid withdrawal and GABAergic inhibition deficits. PPD symptoms are thought to be caused by a deficiency or withdrawal of inhibitory neurosteroids, such as AP, which positively modulate GABA-A receptors, especially extrasynaptic receptors involved in network excitability and anxiety. Other mechanisms could also contribute to the PPD therapeutic response to BX, such as progesterone receptors (Reddy and Mohan 2011), mitigation of stress (Zorumski et al. 2019; Walton and Maguire 2019), and inflammation (Balan et al. 2023).

Despite its many positives, BX therapy also has some constraints. For example, concerns have been raised about the accessibility and cost of BX, which is only available at certified healthcare facilities with REM monitoring and requires a 60-h hospitalization. Under these conditions, only about one-third of women are aware of and have access to BX therapy. Many women face significant barriers to this therapy including lack of time, stigma, and childcare issues during hospitalization. Additionally, the persistence of BX efficacy beyond 30 days is unclear. The lack of an oral pill is a significant barrier that can be overcome by introducing orally active neurosteroids. Further research is needed to address these issues and facilitate the development of effective PPD treatments.

In summary, approval of BX for PPD therapy was a major milestone in neurosteroid research, providing a pathway for further investigation of their therapeutic potential. Neurosteroids offer several advantages over traditional antidepressants, including rapid onset, known mechanism, and the lack of tolerance upon repeated use. Several important restrictions must also be considered, such as limited accessibility, need for hospitalization to receive drug infusion, absence of oral treatment options, and potential for severe side effects at high doses. Neurosteroids could be combined with other medications because of their synergism with benzodiazepines and tiagabine (Chuang and Reddy 2020). Because BX is metabolized by non-CYP enzymes, it has few drug interactions, a highly favorable feature for adjunct therapy. Recently developed, orally active and water-soluble analogs of BX will also support the development of second-generation neurosteroids for PPD.

## Abbreviations

AP: Allopregnanolone
BX: Brexanolone
CNS: Central nervous system
CYP450scc: Cholesterol side-chain cleavage enzyme
HAM-D: Hamilton Depression Rating Scale
HSD: Hydroxysteroid dehydrogenase
HSOR: Hydroxysteroid oxidoreductase
FDA: Food and drug administration
NRT: Neurosteroid replacement therapy
NSW: Neurosteroid withdrawal
PPD: Postpartum depression
SSRI: Selective serotonin reuptake inhibitor
SNRI: Serotonin-norepinephrine reuptake inhibitors
REMS: Risk evaluation and mitigation strategies
